# Application of Quantum Cascade Laser-Infrared Spectroscopy and Chemometrics for In-Line Discrimination of Coeluting Proteins from Preparative Size Exclusion Chromatography

**DOI:** 10.1021/acs.analchem.2c01542

**Published:** 2022-08-04

**Authors:** Christopher K. Akhgar, Julian Ebner, Mirta R. Alcaraz, Julian Kopp, Héctor Goicoechea, Oliver Spadiut, Andreas Schwaighofer, Bernhard Lendl

**Affiliations:** † Institute of Chemical Technologies and Analytics, Technische Universität Wien, Getreidemarkt 9, 1060 Vienna, Austria; ‡ Institute of Chemical, Environmental and Bioscience Engineering, Technische Universität Wien, Getreidemarkt 9, 1060 Vienna, Austria; § Laboratorio de Desarrollo Analítico y Quimiometría (LADAQ), Cátedra de Química Analítica I, Facultad de Bioquímica y Ciencias Biológicas, Universidad Nacional del Litoral, Ciudad Universitaria, S3000ZAA Santa Fe, Argentina; ∥ Consejo Nacional de Investigaciones Científicas y Técnicas (CONICET), Godoy Cruz 2290, C1425FQB CABA, Argentina

## Abstract

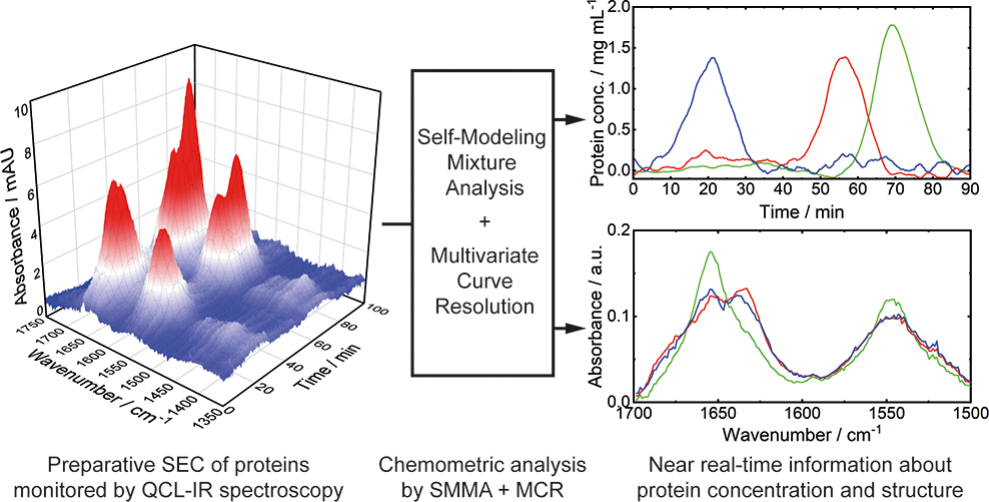

An external-cavity quantum cascade laser (EC-QCL)-based flow-through mid-infrared (IR) spectrometer was placed in line with a preparative size exclusion chromatography system to demonstrate real-time analysis of protein elutions with strongly overlapping chromatographic peaks. Two different case studies involving three and four model proteins were performed under typical lab-scale purification conditions. The large optical path length (25 μm), high signal-to-noise ratios, and wide spectral coverage (1350 to 1750 cm^–1^) of the QCL-IR spectrometer allow for robust spectra acquisition across both the amide I and II bands. Chemometric analysis by self-modeling mixture analysis and multivariate curve resolution enabled accurate quantitation and structural fingerprinting across the protein elution transient. The acquired concentration profiles were found to be in excellent agreement with the off-line high-performance liquid chromatography reference analytics performed on the collected effluent fractions. These results demonstrate that QCL-IR detectors can be used effectively for in-line, real-time analysis of protein elutions, providing critical quality attribute data that are typically only accessible through time-consuming and resource-intensive off-line methods.

Protein purification and polishing protocols typically include diverse process unit operations based on liquid chromatography (LC).^1^ This technique separates analytes in a liquid mobile phase by interactions with a solid stationary phase according to different physio-chemical properties. In size exclusion chromatography (SEC), compounds are separated by their size and shape which offers several advantages, such as straight-forward operation, nondenaturing conditions, and isocratic elution, compared to other separation principles.^2^ Protein concentrations in chromatographic effluents are routinely monitored in-line by univariate UV/vis or evaporative light scattering detectors, offering excellent sensitivity, high robustness, and a broad linear range. A major drawback of these detectors is, however, that the obtained signals do not provide information that allows for the discrimination or quantitation of different coeluting proteins. Critical quality attributes (CQAs), thus, have to be obtained by analyzing the collected fractions off-line. During process development, this can lead to significant time delays. Moreover, it hinders development based on quality by design (QbD) principles. QbD requires the application of process analytical technology (PAT) tools, facilitating in-process monitoring and in-process control. In-line or on-line measurements providing real-time or near real-time information on CQAs are required to allow timely adaption of set-points during the purification step.

Mid-infrared (IR) spectroscopy is a well-established technique for nondestructive analysis of diverse compounds, including polypeptides and proteins.^3^ Conventional Fourier-transform IR (FT-IR) spectrometers are equipped with thermal light sources that emit low-power radiation across the entire mid-IR region (400–4000 cm^–1^). Even though LC-FT-IR hyphenation was successfully demonstrated for the analysis of numerous analytes including nitrophenols,^4,5^ carbohydrates,^6,7^ and pesticides;^8,9^ mid-IR flow-through measurements of proteins remain challenging. The most important IR bands for protein secondary structure determination and quantitation are the amide I (1600–1700 cm^–1^) and amide II (1500–1600 cm^–1^) band, respectively.^10^ Substantial light absorption by the HOH bending band of water at approximately 1645 cm^–1^ makes investigations of the overlapping amide I band with FT-IR instrumentation a cumbersome task. In order to avoid total absorption of IR radiation in this spectral region, optical path lengths of 6 to 8 μm are typically applied.^11,12^ Such limited path lengths are not suitable for LC-IR hyphenation as they lead to distinctly impaired robustness and sensitivity. For this purpose, complex schemes were developed that evaporate the solvent and deposit the protein almost simultaneously onto a substrate before FT-IR analysis.^13^ Even though these setups enabled protein secondary structure analysis from LC effluents,^14,15^ solvent evaporation interfaces can bear major challenges such as spatial heterogeneity and changes in analyte morphology over time.^16^ Moreover, in preparative LC runs, the effluent is usually fractionated after detection, making a preceding solvent evaporation step inapplicable. More recently, attenuated total reflection-FT-IR spectroscopy^17^ was coupled to an LC system for in-line monitoring of proteins.^18−20^ This configuration overcomes the limitations regarding ruggedness, but still requires high protein concentrations due to its limited sensitivity.

Significant progress in quantum cascade lasers (QCLs)^21^ has challenged conventional FT-IR spectrometers for biochemical sensing applications.^22^ Properties such as ≥10^4^ times higher brightness compared to thermal light sources and tunability over several hundred wavenumbers in external cavity (EC) configurations make QCLs highly beneficial for the analysis of proteins.^23^ In this context, diverse academic setups were developed that employed EC-QCLs for protein investigations. Here, it has been demonstrated that the intense power outputs of QCLs allow to significantly increase the path length for transmission measurements and, thus, the ruggedness for protein amide I band analysis in aqueous solutions.^24^ Due to the particular characteristics of water absorption in the protein amide I spectral region and emission properties of EC-QCLs,^25^ it has turned out to be a challenging task to develop setups also covering the protein amide II region. However, simultaneous analyses of amide I + II bands were realized by different approaches, for example, by combining EC-QCLs with either mercury cadmium telluride detectors (MCTs) and optical filters,^25,26^ MCTs and acousto-optic modulators,^27^ or quantum cascade detectors (QCDs).^28^ Furthermore, the implementation of an advanced noise compensation strategy based on balanced detection led to robust protein measurements with limits of detection almost an order of magnitude lower than those from high-end FT-IR spectrometers.^26^

Parallel to the rapid advances of laser-based optical setups in academic research, a commercially available QCL-IR spectrometer, the ChemDetect Analyzer (Daylight Solutions), was recently introduced.^29^ This device covers a broad wavenumber range beyond protein amide I and II bands and offers robust and sensitive spectra acquisition with an optical transmission path of 25 μm. In a recent piece of work, the ChemDetect Analyzer was successfully applied for in-line monitoring of proteins from preparative LC.^30^ Compared to conventionally used LC detectors, laser-based mid-IR spectroscopy offers the major advantage of providing near real-time information about protein quantity and secondary structure, which can otherwise merely be obtained by off-line measurements. LC-QCL-IR coupling, thus, bears a high potential for in-line analysis of CQAs, such as protein purity, which is further investigated in the present study.

For analysis of complex experimental data, chemometrics is typically applied to extract chemical information about individual analytes from spectroscopic data of multicomponent systems. Multivariate spectroscopic monitoring of dynamic processes, such as in LC-QCL-IR, generates two-way data matrices that comprise the information about the occurring spectral changes and the chemical perturbation profiles of the system. Multicomponent spectroscopic signals generally follow Beer–Lambert’s Law and fulfill the concept of the so-called bilinear models.^31^ Among the most used chemometric techniques based on bilinear decomposition are self-modeling mixture analysis (SMMA)^32^ and multivariate curve resolution (MCR).^33^ The advantage of these methods is that they do not require any a priori knowledge about the system, for example, the number or spectra of components, and all information can be deduced from the recorded data set. Even though the obtained pure variables do not represent a pure component, for systems with a reduced number of components, this approach can serve as a good and fast estimator of the chemical behavior of the system that can be readily compared to recorded spectra and, thus, allows straightforward interpretation by nonchemometricians.^34,35^

In this work, LC-QCL-IR hyphenation was performed for in-line monitoring of proteins from coeluting chromatographic peaks. Two case studies involving three and four proteins, respectively, were performed based on SEC, and real-life conditions used in protein purification protocols were applied. The goal of this work is to employ in-line QCL-IR spectroscopy for obtaining qualitative and quantitative information, which can be conventionally only received by work- and time-intensive off-line high-performance LC (HPLC) analytics. For this purpose, chemometric analysis based on a bilinear decomposition model was performed to (i) extract IR absorption spectra of the individual proteins from the recorded multidimensional data set as well as to (ii) retrieve their concentration profiles over the chromatographic run. Achieved results were benchmarked against reference off-line IR spectra of pure protein solutions and HPLC measurements of the collected fractions, showing excellent agreement in both cases.

## Experimental Section

### Reagents and Samples

Ovalbumin (Ova, ≥90%), α-chymotrypsinogen A (α-CT) from bovine pancreas, myoglobin (Myo) from equine skeletal muscle (≥95%), horseradish peroxidase type VI-A (HRP), and β-lactoglobulin (β-LG) from bovine milk (≥90%) were obtained from Sigma-Aldrich (Steinheim, Germany). Appropriate amounts of protein powder were dissolved in SEC buffer. Ultrapure water (MQ) was obtained with a Milli-Q system from Merck Millipore (Darmstadt, Germany). Trifluoroacetic acid and acetonitrile, both HPLC-grade, were purchased from AppliChem (Darmstadt, Germany). All other chemicals used for the preparation of mobile phases were obtained from Carl Roth (Karlsruhe, Germany).

### LC-QCL-IR Flow Path

**Figure 1:**
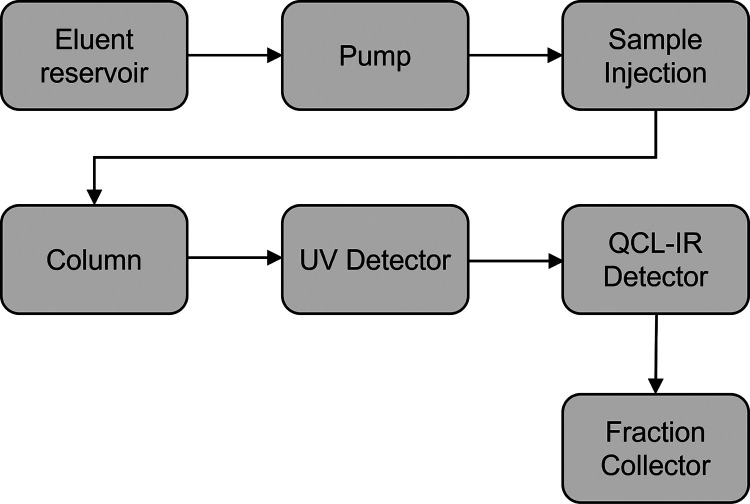
Scheme of the flow path in the LC-IR setup.

The applied LC-QCL-IR setup is depicted in Figure 1. An ÄKTA pure preparative chromatographic system (Cytiva Life Sciences, MA, USA), equipped with a U9-M UV monitor and an F9-C fraction collector was used for all SEC runs. A HiLoad 16/600 Superdex 200 pg (Cytiva Life Sciences, MA, USA) was used as the SEC column for both Case study I and Case study II. A ChemDetect Analyzer (Daylight Solutions Inc., San Diego, USA) was used to record QCL-IR spectra.

### Size Exclusion Chromatography Conditions

For preparative LC runs, the setup described in Figure 1 was used. Both runs were performed in the isocratic mode with a 50 mM phosphate buffer pH 7.4 (SEC buffer) with a constant flow of 7.5 cm/h (=0.25 mL/min). For Case study I, 0.5 mL of SEC buffer containing 10 mg/mL Ova, 10 mg/mL α-CT, and 10 mg/mL Myo were injected. For case study II, 0.5 mL of SEC buffer containing 10 mg/mL HRP, 10 mg/mL β-LG, 10 mg/mL α-CT, and 10 mg/mL Myo were injected. UV absorbance (280 nm) was recorded over the whole run and fractions with a volume of 1 mL were collected. For case study I, the protein concentration of the individual proteins in the collected fractions was measured using reversed-phase (RP) HPLC. For case study II, the concentration of HRP and Myo in the collected fractions were quantified using the described RP-HPLC method, while the concentrations of β-LG and α-CT were obtained using a cation exchange (CEX) HPLC method.

### Laser-Based Mid-IR Measurements

All mid-IR measurements were acquired with a ChemDetect Analyzer. The equipped EC-QCL was operated between 1350 and 1750 cm^–1^ and thermally stabilized with an external water-cooling unit (set to 17 °C). A custom-built, temperature-stabilized CaF_2_ flow cell with an optical path length of 25 μm was used for all transmission measurements. The provided ChemDetect software package was used for spectra acquisition. For LC-QCL-IR in-line measurements, a background spectrum was acquired within 60 s by averaging 121 scans, followed by spectra acquisition every 10 s (averaging of 20 scans). Off-line reference measurements of pure protein solutions were performed by averaging 91 scans within 45 s. During spectra acquisition, the ChemDetect Analyzer was flushed with dry air to decrease the influence of water vapor from the atmosphere.

### HPLC Reference Measurements

As an off-line analytical method to qualify and quantify proteins contained in the collected fractions, a previously published RP-HPLC method was used.^36^ Because it was not possible to achieve satisfactory peak separation for β-LG and α-CT using the RP-HPLC method (case study II), additionally, CEX HPLC measurements were performed. For that purpose, an UltiMate 3000 HPLC system (Thermo Fisher, MA, USA) equipped with a quaternary pump module, a temperature-controlled autosampler, a column oven, and a UV/vis detector module was used. The method used a MabPac SCX-10 (250 mm) column (Thermo Fisher, MA, USA) with a constant column temperature of 35 °C and a constant flow rate of 1 mL/min. In total, three mobile phases (mobile phase A: 20 mM phosphate citrate buffer pH 4; mobile phase B: 20 mM phosphate citrate buffer pH 4 with 1 M NaCl; and mobile phase C: 50 mM phosphate buffer pH 7.4 with 1 M NaCl) were used, and the exact gradient profile is shown in Figure S1. In order to achieve sufficient separation, the pH value of all samples was adjusted to pH 4 (10 M phosphoric acid) prior to the measurement. An injection volume of 20 μL was used for all samples and concentrations were calculated based on peak integration and comparison to measured standards with a known concentration. Standards were treated in the same way as samples, that is, dissolved to the desired concentration in SEC buffer and adjusted to pH 4 using 10 M phosphoric acid.

### Data Analysis

In the present work, the separation of proteins by SEC was monitored with QCL-IR spectroscopy. For data analysis, the spectral range of the data matrix was cut to 1500–1700 cm^–1^, corresponding to the protein amide I and amide II bands, and the temporal range was limited to cover periods of protein elution. Prior to chemometric resolution, aiming to improve the S/N ratio, averaging of two data points was performed in the spectral axis. For case study I, an additional averaging of two spectra was performed in the time axis. Finally, 273 × 174 (case study I) and 271 × 176 (case study II) matrices were obtained and subjected to chemometric analysis.

Multicomponent spectroscopic signals generally follow Beer–Lambert’s Law, hence they fulfill the concept of bilinear models described by
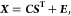
1where ***X*** describes the two-way data matrix, and ***S*** and ***C*** contain the bilinear description of the data for both spectral profile and their relative concentrations, respectively; ***E*** contains the residuals of the model. In the applied workflow, spectral estimates for spectral profiles were obtained by SMMA. This group of techniques estimates the purest chemical factors and their contribution requiring any specific information about the data. In this regard, the pure variable-based methods, such as the simple-to-use interactive SSMA approach (SIMPLISMA), seek to obtain the selective spectral (or concentration) variables through the calculation of a purity value. The subsequently applied MCR, on the other hand, is a family of soft modeling techniques able to solve the bilinear description of the data for both spectral (***S***) profiles and their relative concentrations (***C***) through bilinear decomposition of the two-way data matrix ***X*** either by noniterative or iterative methods.

Data processing and chemometric analysis were performed in MATLAB R2020b (Mathworks, Inc., Natick, MA, 2020). MCR-ALS algorithms are available online at http://www.mcrals.info/.

### Protein Quantitation of Reconstituted Individual LC-QCL-IR Chromatograms

Based on the concentration and spectral profiles obtained by the chemometric analysis, individual chromatogram matrices ***X***_***n***_ (=c_*n*_s_*n*_^T^) were reconstituted for every protein. These reconstituted IR spectra were employed to calculate protein concentrations (*c*) across the chromatographic run according to the Beer–Lambert law
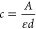
2Here, *d* is the path length of the transmission cell. The absorbance values (*A*) were obtained by integrating the amide II bands (1500–1600 cm^–1^) of the reconstituted QCL-IR spectra. Absorption coefficients (ε) of the selected proteins were obtained by integrating the same spectral region of off-line acquired QCL-IR spectra of reference solutions with known protein concentrations.

## Results and Discussion

To demonstrate the potential of the presented approach, monitoring of preparative SEC by a QCL-IR detector with subsequent chemometric analysis was performed with two-protein model systems exhibiting partial coelution of the proteins.

### Case Study I: A Model System with Three Proteins

**Figure 2:**
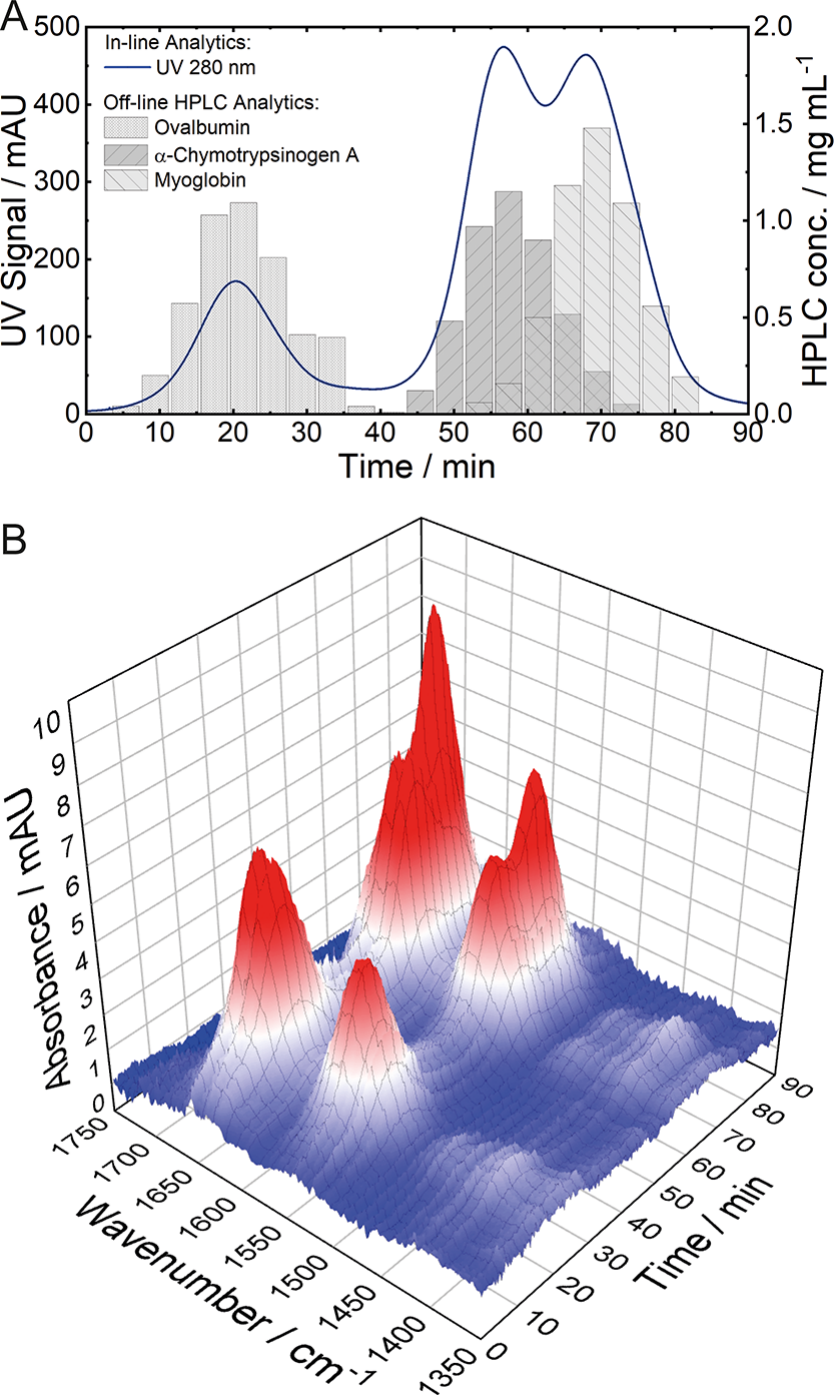
Experimental data obtained from the SEC run of case study I. (A) Results of in-line UV spectroscopy (left) and off-line HPLC analytics (right). (B) Spectral 3D plot recorded by the QCL-IR detector.

For case study I, a SEC run with three different proteins was performed. For this purpose, Ova, α-CT, and Myo were identified to be proteins with different molecular weights and secondary structures. Figure 2A shows the results of in-line UV spectroscopy at 280 nm, indicating three chromatographic peaks. This signal is the most common for protein detection but does not provide any information regarding the secondary structure. Thus, in order to obtain qualitative and quantitative information about the eluting proteins, off-line HPLC analytics need to be performed (Figure 2A). The first chromatographic peak at 22 min can be related to Ova with a molecular weight of 44.5 kDa.^37^ α-CT and Myo have more similar molecular masses of 25.6 kDa^38^ and 17 kDa,^39^ respectively, and show overlapping peaks at approximately 57 and 70 min. These results agree with the separation principle of SEC, where large molecules elute first. The ChemDetect Analyzer was used to record mid-IR spectra of the LC effluent across the chromatographic run.

**Figure 3:**
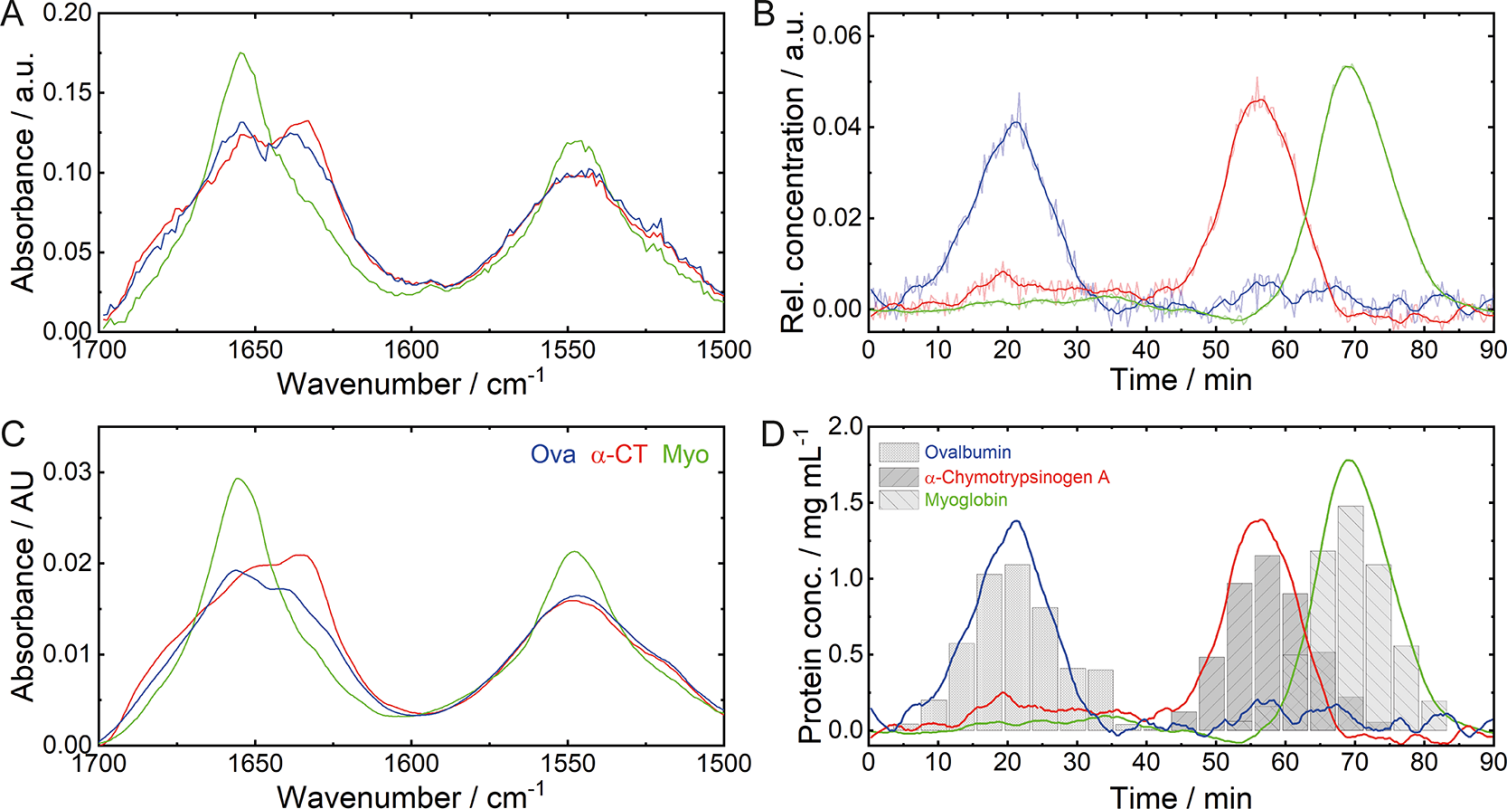
Results obtained by chemometric analysis of the bidimensional QCL-IR data set of case study I. (A) Spectral and (B) time-dependent concentration profiles retrieved by chemometric analysis. The obtained concentration profiles (thin) were smoothed by a Savitzky–Golay filter (thick). (C) Reference laser-based IR spectra of Ova, α-CT, and Myo. (D) Protein concentrations obtained by in-line QCL-IR analysis (lines) and off-line reference HPLC analytics (bars).

Figure 2B displays the 3D plot (wavenumber-time-absorbance) of the performed LC-QCL-IR measurements. The plot shows stable baseline and chromatographic peaks with the characteristic amide I and II bands at retention times corresponding to the three proteins. For rather basic qualitative interpretation and discrimination between the three eluting proteins, chromatograms at wavenumbers characteristic for individual secondary structures can be extracted from the 3D data set and compared.^40^ However, to gain more insight into the qualitative and quantitative information, an in-depth chemometric analysis needed to be performed. For this purpose, first, the number of components was estimated by singular value decomposition. Then, the purest spectral profiles were received by using a SIMPLISMA-like approach.^41^ Subsequently, unconstrained MCR was applied to determine the corresponding time-dependent concentration profiles. At this point, it should be emphasized that for this analysis, no initial knowledge, for example, about the number and type of proteins, is required and all information can be derived from the recorded 3D QCL-IR data set. The obtained lack of fit (LOF, 2.5%) indicates a good description of the experimental data by the MCR model. By this approach, five components were determined to be needed to explain the experimental data, three of them were attributed to the eluting proteins in the chromatographic run, whereas the last two were associated with background signals. Figure 3A,B show the spectral and time-resolved concentration profiles, respectively, of the identified proteins. The retention times at the maximum of the concentration profiles agree very well with the peak maxima observed by reference techniques.

Analysis of the spectral profiles allows assigning secondary structures to the eluted proteins, even if no further reference information is available. The shapes of the amide I and amide II bands indicate that the first two eluting proteins are composed of mixed or β-sheet secondary structures, whereas the third protein mainly contains α-helices. In case reference spectra are available (Figure 3C), identification of the eluted proteins is also possible. Ova features both α-helical and β-sheet secondary structures, resulting in an amide I band maximum at 1656 cm^–1^ with a shoulder at 1638 cm^–1^ and a broad amide II band with the maximum at approximately 1545 cm^–1^.^42,43^ α-CT also contains α-helices but is predominantly composed of β-sheets showing a characteristic broad amide I band with a maximum at 1635 cm^–1^ and shoulders at 1650 and 1680 cm^–1^. The amide II band features a broad shape with a maximum at approximately 1548 cm^–1^.^44^ Even though the spectral profiles of the first and second chromatographic peaks appear similar, evaluation of the different amide I band maxima and bandwidths allows the assignment of the first peak to Ova and the second peak to α-CT. Finally, the third resolved spectral profile can be assigned to Myo, which is mainly composed of an α-helical secondary structure.^45^ The corresponding absorption spectrum shows a distinct amide I band at approximately 1656 cm^–1^ and a narrow amide II band with a maximum at 1547 cm^–1^.

Two additional components obtained from the chemometric analysis were identified in the recorded QCL-IR data set (Figure S2). One concentration profile featured negative dips at the same retention times as the chromatographic protein peaks. Thus, this profile was assigned to the dilution of the buffer during protein elution, as the presence of proteins reduces the relative water content as compared to the background spectrum (pure buffer). Thus, due to the high absorption coefficient of the HOH-bending band, even subtle variations of the water content can introduce an observable effect on the IR spectra.^46^ One further component was assigned to varying baseline and instrumental responses.

### Case Study II: A Model System with Four Proteins

**Figure 4:**
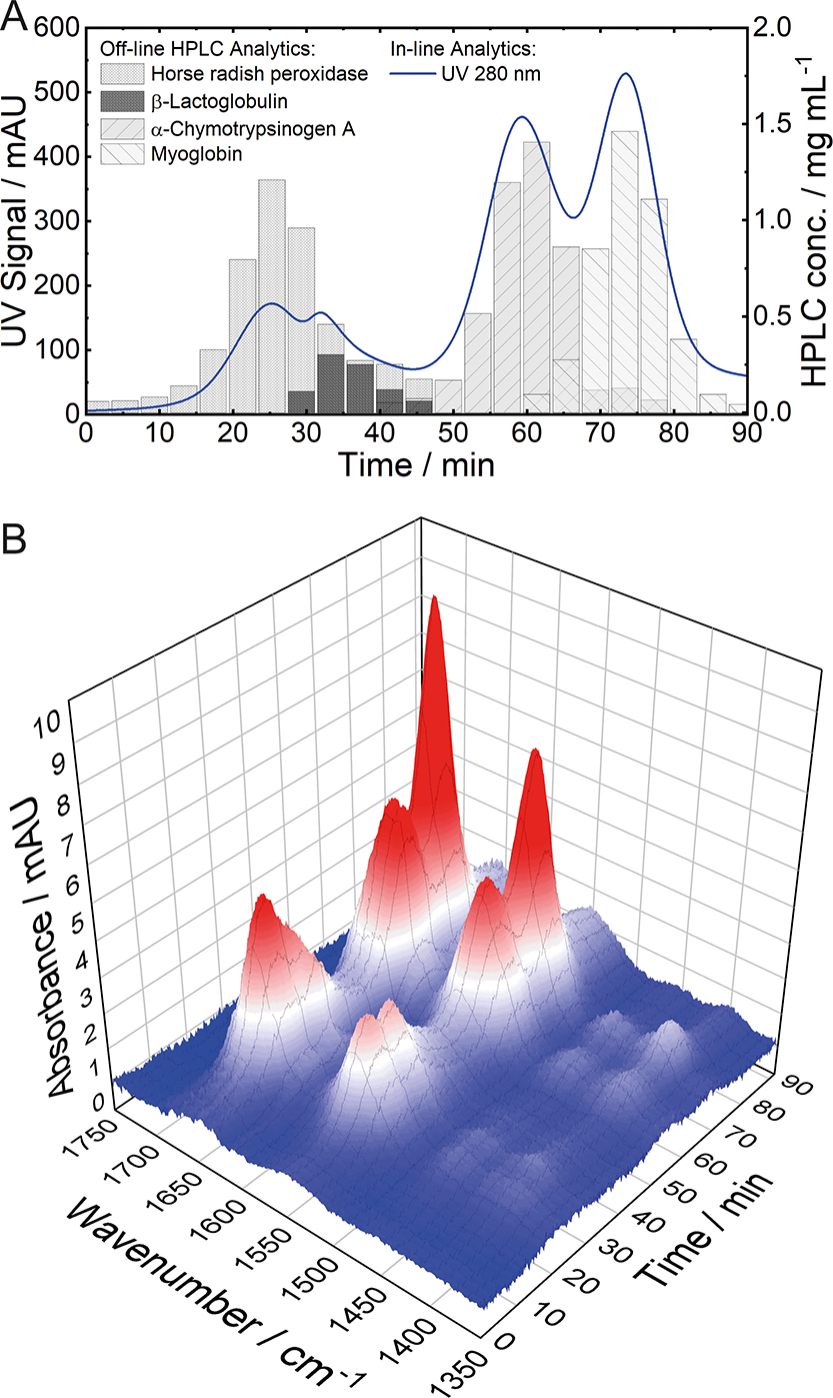
Experimental data obtained from the SEC run of case study II. (A) Results of in-line UV spectroscopy (left) and off-line HPLC analytics (right). (B) Spectral 3D plot recorded by the QCL-IR detector.

After the successful application of QCL-IR spectroscopy combined with chemometric analysis for protein structure identification and resolving of individual protein chromatograms with the model system comprising three proteins, a further, even more challenging case study was devised to validate the potential and versatility of the introduced method. To this end, a SEC run including HRP, β-LG, α-CT, and Myo was performed. Figure 4A shows the results of in-line UV spectroscopy at 280 nm as well as off-line HPLC analytics, indicating four chromatographic peaks with a severe overlap of the first two protein peaks at 25 and 32 min. The first of these chromatographic peaks can be attributed to HRP with the highest molecular weight of 44 kDa.^47^ The second peak is related to β-LG, which is present at the employed pH conditions in its dimeric form with a molecular mass of 36.7 kDa.^48^ Due to the similar masses of these two proteins, they show highly coeluting behavior. Finally, the two remaining peaks at 59 and 72 min are assigned to α-CT and Myo with molecular masses of 25.6^38^ and 17 kDa,^39^ respectively.

**Figure 5:**
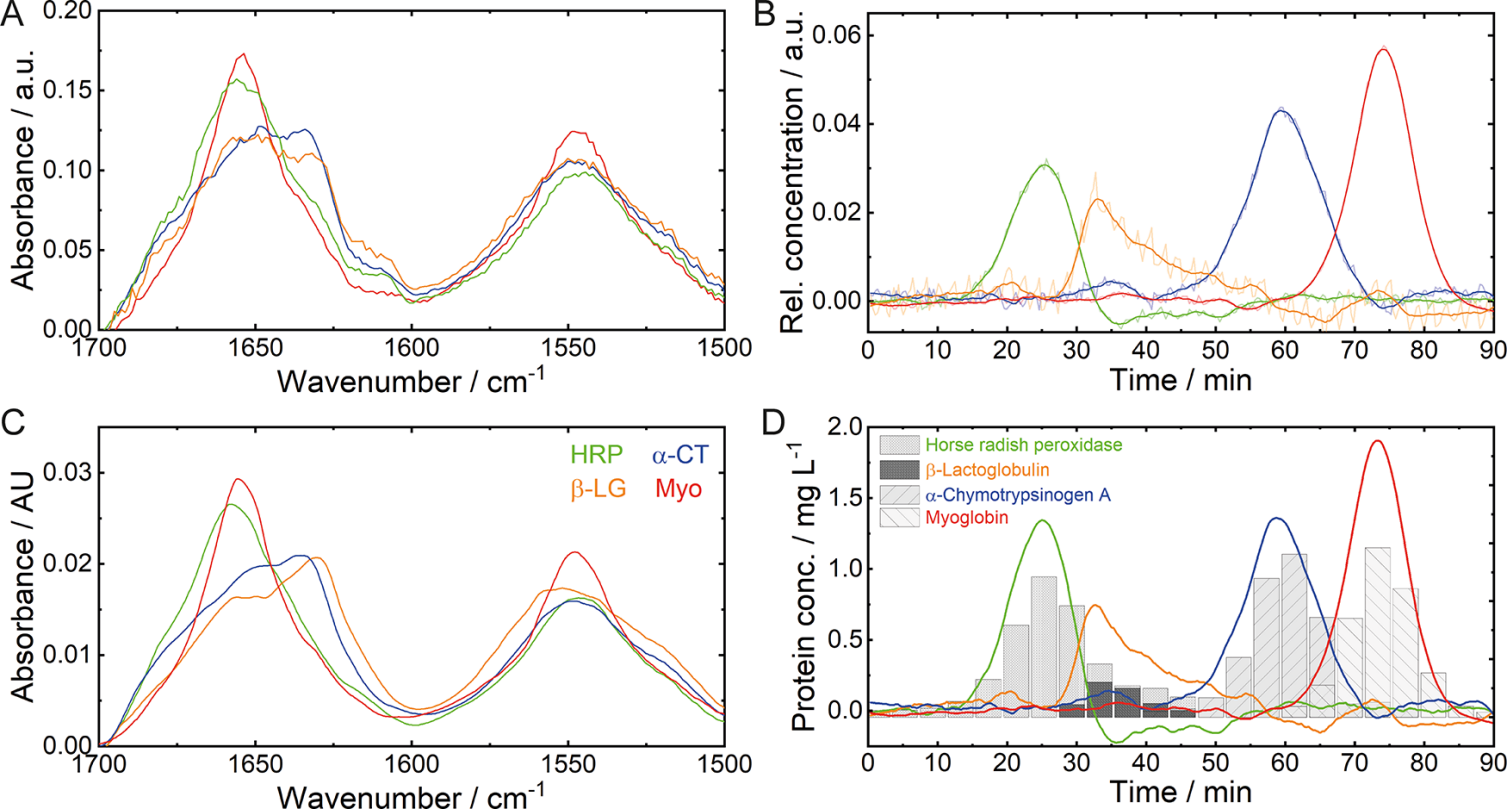
Results obtained by chemometric analysis of the bidimensional QCL-IR data set of case study II. (A) Spectral and (B) time-dependent concentration profiles retrieved by chemometric analysis. The obtained concentration profiles (thin) were smoothed by a Savitzky–Golay filter (thick). (C) Reference laser-based IR spectra of HRP, β-LG, α-CT, and Myo. (D) Protein concentrations obtained by in-line QCL-IR analysis (lines) and off-line reference HPLC analytics (bars).

Figure 4B shows the 3D plot (wavenumber-time-absorbance) of the performed LC-QCL-IR measurement. This plot shows amide I and amide II maxima at retention times comparable with the reference techniques. Also, in this case, the data set was subjected to chemometric analysis to retrieve spectral and time-dependent concentration profiles of the eluting proteins. After obtaining initial estimates, MCR was performed obtaining an LOF of 2.3%, indicating a good fit of the MCR model to the experimental data. For this data set, six components were identified of which four could be assigned to proteins in the chromatographic effluent. The spectral and time-resolved concentration profiles of the identified proteins are shown in Figure 5A,B. The maximum positions of the concentration profiles agree very well with the retention times observed by the reference techniques. Without any prior knowledge, the secondary structures of the first and fourth eluting proteins could be assigned to be mostly α-helical, while the second and third eluting proteins show spectral features of a mixed or β-sheet secondary structure. Comparison with the reference spectra allows identification of the first chromatographic peak as HRP with an amide I band maximum at 1656 cm^–1^ with shoulders at 1640 and 1680 cm^–1^ and an amide II band maximum at approximate 1545 cm^–1^.^49,50^ It can be distinguished from Myo due to the slightly shifted amide I band maximum and the overall broader shape. The spectral profiles of the second and third identified chromatographic peaks have very similar shapes. However, comparison with reference spectra allows identifying the second peak as β-LG due to the narrower amide I band and the broader amide II band shapes. β-LG is predominantly composed of β-sheet secondary structures and shows a distinct amide I band with a maximum at 1632 cm^–1^ and a shoulder at 1660 cm^–1^ and a broad amide II band with a maximum at 1550 cm^–1^.^51,52^ Finally, the spectral profile of the fourth chromatographic peak can be unanimously attributed to Myo.

Evaluation of the concentration profile reveals negative relative concentrations of HRP at retention times between 30 and 50 min. Impaired resolution of the bidimensional data set by MCR in this time region is caused by the severe overlap in retention times of HRP and β-LG as also shown by the reference HPLC results. Furthermore, α-CT which also starts to elute in this period features similar IR spectral features as β-LG.

Chemometric analysis of this data set further identified two additional components (Figure S3), as occurred for case study I. Due to the shape of the concentration profiles, one was attributed to varying baseline and buffer dilution in the presence of proteins. The concentration profile of the other component shows a zigzag shape at rather low relative concentrations. This periodic noise characteristic is partly also visible in the concentration profile of β-LG. It is present in the IR measurements, but not observable in the in-line UV measurements. The origin of these features was traced back to inconstant dry air supply throughout the QCL-IR measurements. The periodic behavior is introduced by a switching valve in the purge gas generator. Furthermore, the involvement of IR absorption of water vapor in this repetitive noise pattern is also supported by the narrow absorption bands in the related spectral profile.

Notwithstanding this instrumental issue, owing to the challenging model system and experimental difficulties, it was decided to present these results as proof to demonstrate the potency of the presented workflow to obtain reliable estimations by the combination of QCL-IR spectroscopy and chemometrics.

### In-Line Protein Quantitation by QCL-IR Spectroscopy

The reconstituted IR spectra obtained from chemometric analysis were employed for the calculation of the absolute protein concentrations. For this purpose, the area of the amide II band was integrated because this spectral region is less prone to water absorption-related intensity variations than the amide I band. Absorption coefficients of the proteins included in case studies I and II were obtained from QCL-IR off-line measurements with known protein concentrations. Figure 3D shows a comparison between the calculated concentrations based on QCL-IR spectroscopy and reference HPLC results of the collected fractions for case study I. The graph demonstrates highly overlapping concentration profiles between in-line and off-line reference measurements, indicating high validity of the presented approach based on QCL-IR spectroscopy and chemometrics. This evaluation was also performed for case study II (Figure 5D). Here, the elution profiles of all four proteins agree well between the two methods, even though absolute concentrations appear slightly shifted. These differences might be explained by the highly overlapping spectral features of β-LG and α-CT and the pronounced overlap of the chromatographic peaks of HRP and β-LG. Those challenging circumstances may adversely affect chemometric analysis and lead to underestimation of the HRP content at retention times between 30 and 50 min while overestimating the β-LG content. Nevertheless, these good results, in spite of the complex data sets, indicate high flexibility and robustness of the presented LC-QCL-IR approach.

Case study II also was challenging to resolve for off-line RP-HPLC analytics. The insufficient peak resolution of β-LG and α-CT required the introduction of an additional CEX-HPLC method in order to accurately identify and quantify the proteins in the collected fractions. As two different off-line HPLC methods are required to analyze fractions in case study II, this further emphasizes the difficulties to establish straightforward reference analytics. Consequently, QCL-IR in-line detectors hold significant potential for providing near-real-time protein concentrations from chromatographic separation processes by achieving similar results as conventionally applied time- and cost-intensive off-line methods.

## Conclusions and Outlook

In this work, an EC-QCL-based mid-IR spectrometer was successfully hyphenated to a preparative SEC system for in-line discrimination of proteins from highly overlapping chromatographic peaks. The advantages of QCL-IR detectors over conventional LC detectors were demonstrated in two case studies, involving mixtures of three and four different proteins, respectively. Due to similar molecular weights of the proteins, highly overlapping chromatographic peaks were obtained that could not be distinguished with a standard UV detector. In contrast, QCL-IR detection enabled the acquisition of multivariate data sets, containing mid-IR absorbance spectra across the chromatographic runs that provide information regarding protein secondary structure, thus allowing protein identification. These data sets were investigated by chemometrics to obtain spectral profiles of the individual proteins as well as their relative concentration profiles. The obtained spectra agree well with reference off-line spectra of pure protein solutions. Furthermore, absolute protein concentrations were calculated according to the Beer–Lambert law, showing high agreement with HPLC reference measurements of the collected effluent fractions. Consequently, QCL-IR in-line detectors can provide qualitative and quantitative information about proteins, comparable to time- and labor-intensive off-line methods that are inaccessible with conventional UV detectors. Hence, the in-line QCL-IR system has the capability to (i) accelerate process development and to (ii) monitor production processes on-line, allowing in-process control. Furthermore, the presented system enables QbD principles and concurs with the requirements of a PAT tool, providing information about protein secondary structure in real-time.

Finally, an important property of the presented in-line QCL-IR detection of preparative LC is its accordance with green analytical chemistry (GAC) principles.^53^ For a comprehensive comparison of in-line QCL-IR spectroscopy and off-line HPLC analysis, the previously introduced Analytical GREEnness (AGREE) metric approach,^54^ a straightforward assessment approach based on the 12 principles of GAC (SIGNIFICANCE),^55^ was applied. The results for both methods are shown in Figure S4. The scores of 0.84 for QCL-IR spectroscopy and 0.43 for off-line HPLC indicate a clear superiority of the presented in-line QCL-IR method in terms of the greenness of the analytical procedure.

## Supporting Information Available

Gradient profile used in the CEX-HPLC method; additional spectra and time-resolved concentration profiles retrieved by chemometric analysis of the chromatographic run of case study I and case study II; results of evaluation according to the Analytical GREEnness metric approach for in-line QCL-IR spectroscopy and off-line HPLC analysis; and selected options for the Analytical GREEnness evaluation are presented here (PDF)

## References

[ref1] Deutscher M. P. Chapter 5 Setting Up a Laboratory. In Methods in Enzymology; Burgess R. R., Deutscher M. P., Eds.; Academic Press, 2009; pp 37–42. 10.1016/S0076-6879(09)63005-6 19892165

[ref2] Sun Y.; Shi Q. H.; Zhang L.; Zhao G. F.; Liu F. F. 2.47—Adsorption and Chromatography. In Comprehensive Biotechnology (2nd ed.), Moo-Young M., Ed.; Academic Press: Burlington, 2011; pp 665–679.

[ref3] Barth A. Infrared spectroscopy of proteins. Biochim. Biophys. Acta, Bioenerg. 2007, 1767, 1073–1101. 10.1016/j.bbabio.2007.06.004. 17692815

[ref4] Quintás G.; Kuligowski J.; Lendl B. On-Line Fourier Transform Infrared Spectrometric Detection in Gradient Capillary Liquid Chromatography Using Nanoliter-Flow Cells. Anal. Chem. 2009, 81, 3746–3753. 10.1021/ac8025459. 19382774

[ref5] Kuligowski J.; Quintás G.; Garrigues S.; de la Guardia M. Application of point-to-point matching algorithms for background correction in on-line liquid chromatography–Fourier transform infrared spectrometry (LC–FTIR). Talanta 2010, 80, 1771–1776. 10.1016/j.talanta.2009.10.021. 20152409

[ref6] Kuligowski J.; Quintás G.; Garrigues S.; de la Guardia M. Determination of critical eluent composition for polyethylenglycols using on-line liquid chromatography—Fourier transform infrared spectrometry. Anal. Chim. Acta 2008, 624, 278–285. 10.1016/j.aca.2008.06.055. 18706334

[ref7] Kuligowski J.; Quintás G.; Garrigues S.; de la Guardia M. New background correction approach based on polynomial regressions for on-line liquid chromatography–Fourier transform infrared spectrometry. J. Chromatogr. A 2009, 1216, 3122–3130. 10.1016/j.chroma.2009.01.110. 19232625

[ref8] Quintás G.; Lendl B.; Garrigues S.; de la Guardia M. Univariate method for background correction in liquid chromatography–Fourier transform infrared spectrometry. J. Chromatogr. A 2008, 1190, 102–109. 10.1016/j.chroma.2008.02.111. 18378257

[ref9] Quintás G.; Kuligowski J.; Lendl B. Procedure for automated background correction in flow systems with infrared spectroscopic detection and changing liquid-phase composition. Appl. Spectrosc. 2009, 63, 1363–1369. 10.1366/000370209790108914. 20030981

[ref10] Singh B. R. Basic Aspects of the Technique and Applications of Infrared Spectroscopy of Peptides and Proteins. Infrared Analysis of Peptides and Proteins; American Chemical Society: Washington D.C., USA, 1999; pp 2–37.

[ref11] Fabian H.; Mäntele W. Infrared Spectroscopy of Proteins. In Handbook of Vibrational Spectroscopy; John Wiley & Sons: Hoboken, USA, 2006.

[ref12] Yang H.; Yang S.; Kong J.; Dong A.; Yu S. Obtaining information about protein secondary structures in aqueous solution using Fourier transform IR spectroscopy. Nat. Protoc. 2015, 10, 382–396. 10.1038/nprot.2015.024. 25654756

[ref13] Turula V.; de Haseth J. Evaluation of Particle Beam Fourier Transform Infrared Spectrometry for the Analysis of Globular Proteins: Conformation of β-Lactoglobulin and Lysozyme. Appl. Spectrosc. 1994, 48, 1255–1264. 10.1366/0003702944027390.

[ref14] Turula V. E.; de Haseth J. A. Particle Beam LC/FT-IR Spectrometry Studies of Biopolymer Conformations in Reversed-Phase HPLC Separations: Native Globular Proteins. Anal. Chem. 1996, 68, 629–638. 10.1021/ac950874h.

[ref15] Turula V.; Bishop R.; Ricker R.; de Haseth J. Complete structure elucidation of a globular protein by particle beam liquid chromatography - Fourier transform infrared spectrometry and electrospray liquid chromatography-mass spectrometry. Sequence and conformation of β-lactoglobulin. J. Chromatogr. A 1997, 763, 91–103. 10.1016/s0021-9673(96)00960-0. 9129318

[ref16] Kuligowski J.; Quintas G.; Guardia M.; Lendl B. Liquid Chromatography—Liquid Chromatography–Fourier Transform Infrared. Encyclopedia of Analytical Science, 3rd ed.; Elsevier: Amsterdam, Netherlands, 2019; pp 75–85.

[ref17] Ramer G.; Lendl B. Attenuated Total Reflection Fourier Transform Infrared Spectroscopy. In Encyclopedia of Analytical Chemistry; Meyers R., Meyers R., Eds.; John Wiley & Sons: Hoboken, USA, 2013.

[ref18] Großhans S.; Rüdt M.; Sanden A.; Brestrich N.; Morgenstern J.; Heissler S.; Hubbuch J. In-line Fourier-Transform Infrared Spectroscopy as a Versatile Process Analytical Technology for Preparative Protein Chromatography. J. Chromatogr. A 2018, 1547, 37–44. 10.1016/j.chroma.2018.03.005. 29530404

[ref19] Sanden A.; Suhm S.; Rüdt M.; Hubbuch J. Fourier-transform infrared spectroscopy as a process analytical technology for near real time in-line estimation of the degree of PEGylation in chromatography. J. Chromatogr. A 2019, 1608, 460410 10.1016/j.chroma.2019.460410. 31395360

[ref20] Walch N.; Scharl T.; Felföldi E.; Sauer D. G.; Melcher M.; Leisch F.; Dürauer A.; Jungbauer A. Prediction of the Quantity and Purity of an Antibody Capture Process in Real Time. Biotechnol. J. 2019, 14, e1800521 10.1002/biot.201800521. 30945440

[ref21] Faist J.; Capasso F.; Sivco D.; Sirtori C.; Hutchinson A.; Cho A. Quantum Cascade Laser. Science 1994, 264, 553–556. 10.1126/science.264.5158.553. 17732739

[ref22] Schwaighofer A.; Brandstetter M.; Lendl B. Quantum cascade lasers (QCLs) in biomedical spectroscopy. Chem. Soc. Rev. 2017, 46, 5903–5924. 10.1039/c7cs00403f. 28816307

[ref23] Schwaighofer A.; Lendl B. Quantum cascade laser-based infrared transmission spectroscopy of proteins in solution. In Vibrational Spectroscopy in Protein Research; Ozaki Y., Baranska M., Lednev I., Wood B., Eds.; Academic Press: Cambridge, USA, 2020; pp 59–88.

[ref24] Alcaráz M. R.; Schwaighofer A.; Kristament C.; Ramer G.; Brandstetter M.; Goicoechea H.; Lendl B. External-Cavity Quantum Cascade Laser Spectroscopy for Mid-IR Transmission Measurements of Proteins in Aqueous Solution. Anal. Chem. 2015, 87, 6980–6987. 10.1021/acs.analchem.5b01738. 26059222

[ref25] Schwaighofer A.; Montemurro M.; Freitag S.; Kristament C.; Culzoni M. J.; Lendl B. Beyond Fourier Transform Infrared Spectroscopy: External Cavity Quantum Cascade Laser-Based Mid-infrared Transmission Spectroscopy of Proteins in the Amide I and Amide II Region. Anal. Chem. 2018, 90, 7072–7079. 10.1021/acs.analchem.8b01632. 29762006

[ref26] Akhgar C. K.; Ramer G.; Żbik M.; Trajnerowicz A.; Pawluczyk J.; Schwaighofer A.; Lendl B. The Next Generation of IR Spectroscopy: EC-QCL-Based Mid-IR Transmission Spectroscopy of Proteins with Balanced Detection. Anal. Chem. 2020, 92, 9901–9907. 10.1021/acs.analchem.0c01406. 32597635 PMC7376528

[ref27] Chon B.; Xu S.; Lee Y. J. Compensation of Strong Water Absorption in Infrared Spectroscopy Reveals the Secondary Structure of Proteins in Dilute Solutions. Anal. Chem. 2021, 93, 2215–2225. 10.1021/acs.analchem.0c04091. 33433190 PMC8274434

[ref28] Dabrowska A.; David M.; Freitag S.; Andrews A. M.; Strasser G.; Hinkov B.; Schwaighofer A.; Lendl B. Broadband laser-based mid-infrared spectroscopy employing a quantum cascade detector for milk protein analysis. Sens. Actuators, B 2022, 350, 130873 10.1016/j.snb.2021.130873.

[ref29] Schwaighofer A.; Akhgar C. K.; Lendl B. Broadband laser-based mid-IR spectroscopy for analysis of proteins and monitoring of enzyme activity. Spectrochim. Acta, Part A 2021, 253, 119563 10.1016/j.saa.2021.119563. 33621933

[ref30] Akhgar C. K.; Ebner J.; Spadiut O.; Schwaighofer A.; Lendl B. QCL–IR Spectroscopy for In-Line Monitoring of Proteins from Preparative Ion-Exchange Chromatography. Anal. Chem. 2022, 94, 5583–5590. 10.1021/acs.analchem.1c05191. 35353485 PMC9008697

[ref31] de Juan A.; Jaumot J.; Tauler R. Multivariate Curve Resolution (MCR). Solving the mixture analysis problem. Anal. Methods 2014, 6, 4964 10.1039/c4ay00571f.

[ref32] Kucheryavskiy S.; Windig W.; Bogomolov A. Chapter 3—Spectral Unmixing Using the Concept of Pure Variables. In Data Handling in Science and Technology; Ruckebusch C., Ed.; Elsevier, 2016; pp 53–99.

[ref33] Tauler R. Multivariate curve resolution applied to second order data. Chemom. Intell. Lab. Syst. 1995, 30, 133–146. 10.1016/0169-7439(95)00047-x.

[ref34] Hu B.; Sun D.-W.; Pu H.; Wei Q. Rapid nondestructive detection of mixed pesticides residues on fruit surface using SERS combined with self-modeling mixture analysis method. Talanta 2020, 217, 120998 10.1016/j.talanta.2020.120998. 32498854

[ref35] Mansoldo F. R. P.; Berrino E.; Guglielmi P.; Carradori S.; Carta F.; Secci D.; Supuran C. T.; Vermelho A. B. An innovative spectroscopic approach for qualitative and quantitative evaluation of Mb-CO from myoglobin carbonylation reaction through chemometrics methods. Spectrochim. Acta, Part A 2022, 267, 120602 10.1016/j.saa.2021.120602. 34801390

[ref36] Kopp J.; Zauner F. B.; Pell A.; Hausjell J.; Humer D.; Ebner J.; Herwig C.; Spadiut O.; Slouka C.; Pell R. Development of a generic reversed-phase liquid chromatography method for protein quantification using analytical quality-by-design principles. J. Pharm. Biomed. Anal. 2020, 188, 113412 10.1016/j.jpba.2020.113412. 32590301

[ref37] Strixner T.; Kulozik U. 7—Egg proteins. In Handbook of Food Proteins; Phillips G. O., Williams P. A., Eds.; Woodhead Publishing, 2011; pp 150–209.

[ref38] Wilcox P. E. [5] Chymotrypsinogens—chymotrypsins. Methods in Enzymology; Academic Press, 1970; pp 64–108.

[ref39] Zaia J.; Annan R. S.; Biemann K. The correct molecular weight of myoglobin, a common calibrant for mass spectrometry. Rapid Commun. Mass Spectrom. 1992, 6, 32–36. 10.1002/rcm.1290060108. 1591398

[ref40] Akhgar C.; Ebner J.; Spadiut O.; Schwaighofer A.; Lendl B. Laser-based mid-infrared spectroscopy enables in-line detection of protein secondary structure from preparative liquid chromatography. Biomedical Vibrational Spectroscopy 2022: Advances in Research and Industry; SPIE, 2022; Vol. 11957.

[ref41] Windig W.; Bogomolov A.; Kucheryavskiy S. Two-Way Data Analysis: Detection of Purest Variables. In Comprehensive Cheometircs: Chemical and Biochemical Data Analysis; Brown S., Tauler R., Walczak B., Eds.; Elsevier, 2020; pp 275–307.

[ref42] Stein P. E.; Leslie A. G. W.; Finch J. T.; Turnell W. G.; McLaughlin P. J.; Carrell R. W. Crystal structure of ovalbumin as a model for the reactive centre of serpins. Nature 1990, 347, 99–102. 10.1038/347099a0. 2395463

[ref43] Dong A.; Meyer J. D.; Brown J. L.; Manning M. C.; Carpenter J. F. Comparative Fourier Transform Infrared and Circular Dichroism Spectroscopic Analysis of α1-Proteinase Inhibitor and Ovalbumin in Aqueous Solution. Arch. Biochem. Biophys. 2000, 383, 148–155. 10.1006/abbi.2000.2054. 11097188

[ref44] Freer S. T.; Kraut J.; Robertus J. D.; Wright H. T.; Nguyen-Huu-Xuong X. Chymotrypsinogen: 2,5-Å crystal structure, comparison with α-chymotrypsin, and implications for zymogen activation. Biochemistry 1970, 9, 1997–2009. 10.1021/bi00811a022. 5442169

[ref45] Kendrew J. C.; Bodo G.; Dintzis H. M.; Parrish R. G.; Wyckoff H.; Phillips D. C. A Three-Dimensional Model of the Myoglobin Molecule Obtained by X-Ray Analysis. Nature 1958, 181, 662–666. 10.1038/181662a0. 13517261

[ref46] Kuligowski J.; Schwaighofer A.; Alcaráz M. R.; Quintás G.; Mayer H.; Vento M.; Lendl B. External cavity-quantum cascade laser (EC-QCL) spectroscopy for protein analysis in bovine milk. Anal. Chim. Acta 2017, 963, 99–105. 10.1016/j.aca.2017.02.003. 28335981

[ref47] Wisdom G. B. Horseradish Peroxidase Labeling of IgG Antibody. In The Protein Protocols Handbook; Walker J. M., Ed.; Humana Press: Totowa, NJ, 2009; pp 681–683.

[ref48] Madureira A. R.; Pereira C. I.; Gomes A. M. P.; Pintado M. E.; Xavier Malcata F. Bovine whey proteins—Overview on their main biological properties. Food Res. Int. 2007, 40, 1197–1211. 10.1016/j.foodres.2007.07.005.

[ref49] Gajhede M.; Schuller D. J.; Henriksen A.; Smith A. T.; Poulos T. L. Crystal structure of horseradish peroxidase C at 2.15 Å resolution. Nat. Struct. Mol. Biol. 1997, 4, 1032–1038. 10.1038/nsb1297-1032. 9406554

[ref50] Tavares T. S.; da Rocha E. P.; Esteves Nogueira F. G.; Torres J. A.; Silva M. C.; Kuca K.; Ramalho T. C. Δ-FeOOH as Support for Immobilization Peroxidase: Optimization via a Chemometric Approach. Molecules 2020, 25, 259 10.3390/molecules25020259. PMC7024332 31936386

[ref51] Dousseau F.; Pezolet M. Determination of the Secondary Structure Content of Proteins in Aqueous Solutions from Their Amide I and Amide II Infrared Bands. Comparison between Classical and Partial Least-Squares Methods. Biochemistry 1990, 29, 8771–8779. 10.1021/bi00489a038. 2271555

[ref52] Monaco H. L.; Zanotti G.; Spadon P.; Bolognesi M.; Sawyer L.; Eliopoulos E. E. Crystal structure of the trigonal form of bovine beta-lactoglobulin and of its complex with retinol at 2.5 Å resolution. J. Mol. Biol. 1987, 197, 695–706. 10.1016/0022-2836(87)90476-1. 3430598

[ref53] Armenta S.; Garrigues S.; de la Guardia M. Green Analytical Chemistry. Trends Anal. Chem. 2008, 27, 497–511. 10.1016/j.trac.2008.05.003.

[ref54] Pena-Pereira F.; Wojnowski W.; Tobiszewski M. AGREE—Analytical GREEnness Metric Approach and Software. Anal. Chem. 2020, 92, 10076–10082. 10.1021/acs.analchem.0c01887. 32538619 PMC7588019

[ref55] Gałuszka A.; Migaszewski Z.; Namieśnik J. The 12 principles of green analytical chemistry and the SIGNIFICANCE mnemonic of green analytical practices. Trends Anal. Chem. 2013, 50, 78–84. 10.1016/j.trac.2013.04.010.

