# Supplementary material for: Application of Quantum Cascade Laser-Infrared Spectroscopy and Chemometrics for In-Line Discrimination of Coeluting Proteins from Preparative Size Exclusion Chromatography
Source: Anal Chem. 2022 Aug 4;94(32):11192–200. doi: 10.1021/acs.analchem.2c01542 (PMC9386682; doi:10.1021/acs.analchem.2c01542)
Supplement: Supplementary file 1 — ac2c01542_si_001.pdf [file ac2c01542_si_001.pdf]

Supporting information

Application of QCL-IR Spectroscopy and Chemometrics for In-line Discrimination of Co-eluting Proteins from Preparative Size Exclusion Chromatography

Christopher K. Akhgar,[†,‡] Julian Ebner,[§,‡] Mirta R. Alcaraz,[⊥,∥] Julian Kopp,[§] Héctor Goicoechea,[⊥,∥] Oliver Spadiut,[§] Andreas Schwaighofer*[,†] and Bernhard Lendl*[,†]

[†]Institute of Chemical Technologies and Analytics, Technische Universität Wien, Getreidemarkt 9, 1060 Vienna, Austria

[§]Institute of Chemical, Environmental and Bioscience Engineering, Technische Universität Wien, Getreidemarkt 9, 1060 Vienna, Austria

[⊥]Laboratorio de Desarrollo Analítico y Quimiometría (LADAQ), Cátedra de Química Analítica I, Facultad de Bioquímica y Ciencias Biológicas, Universidad Nacional del Litoral, Ciudad Universitaria, Santa Fe, S3000ZAA, Argentina

[∥]Consejo Nacional de Investigaciones Científicas y Técnicas (CONICET), Godoy Cruz 2290, CABA, C1425FQB, Argentina

Figure S1. Exact gradient profile of the used CEX-HPLC method.

Figure S2. Additional spectra and time-resolved concentration profiles retrieved by chemometric analysis of chromatographic run of case study I.

Figure S3. Additional spectra and time-resolved concentration profiles retrieved by chemometric analysis of chromatographic run of case study II.

Figure S4. Results of evaluation according to the Analytical GREEnness metric approach for in-line QCL-IR spectroscopy and off-line HPLC analysis.

Table S1. Selected options for the Analytical GREEnness evaluation.

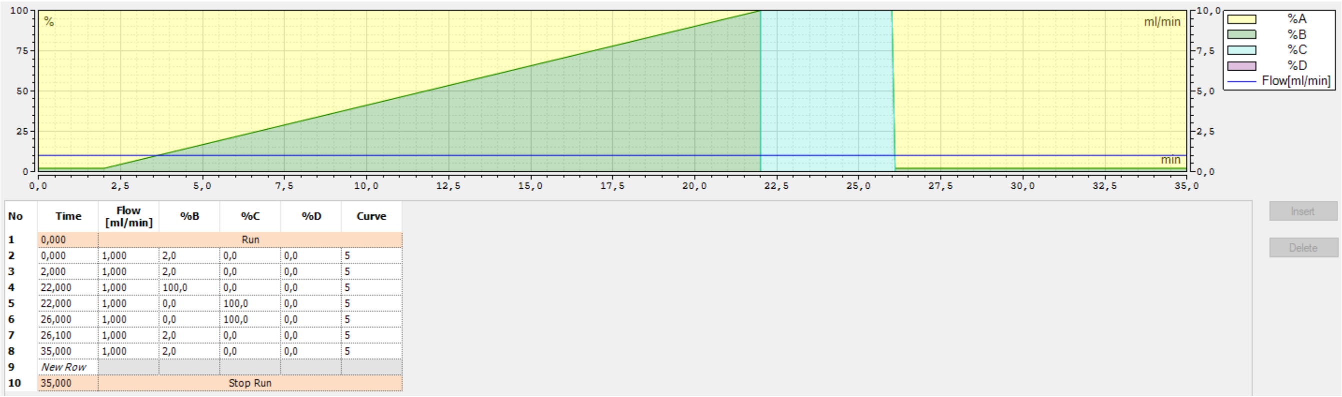

No	Time	Flow [ml/min]	%B	%C	%D	Curve
1	0,000	Run				
2	0,000	1,000	2,0	0,0	0,0	5
3	2,000	1,000	2,0	0,0	0,0	5
4	22,000	1,000	100,0	0,0	0,0	5
5	22,000	1,000	0,0	100,0	0,0	5
6	26,000	1,000	0,0	100,0	0,0	5
7	26,100	1,000	2,0	0,0	0,0	5
8	35,000	1,000	2,0	0,0	0,0	5
9	New Row					
10	35,000	Stop Run				

Figure S1. Exact gradient profile used for the CEX HPLC method with the following mobile phases: mobile phase A: 20 mM phosphate citrate buffer pH 4; mobile phase B: 20 mM phosphate citrate buffer pH 4 with 1 M NaCl; mobile phase C: 50 mM phosphate buffer pH 7.4 with 1 M NaCl. The flow was kept constant at 1 mL/min for the whole run.

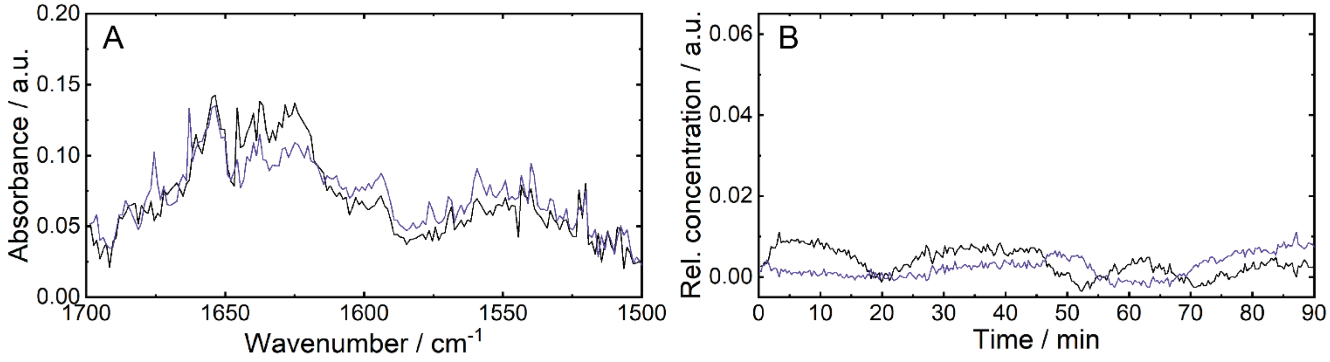

Figure S2. Additional (A) spectra and (B) time-resolved concentration profile retrieved from chemometric analysis of the chromatographic run of case study I. The black component is attributed to dilution effects during protein elution and the violet component is assigned to baseline drifts.

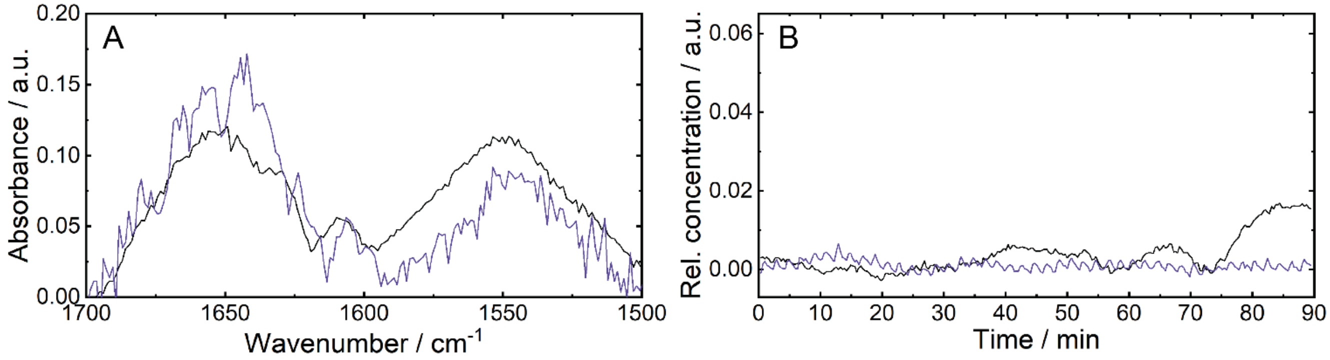

Figure S3. Additional (A) spectra and (B) time-resolved concentration profile retrieved from the chemometric analysis of chromatographic run of case study II. One component (black) component is attributed to dilution effects during protein elution and baseline drifts, while the other component (violet) is assigned to instrumental perturbations.

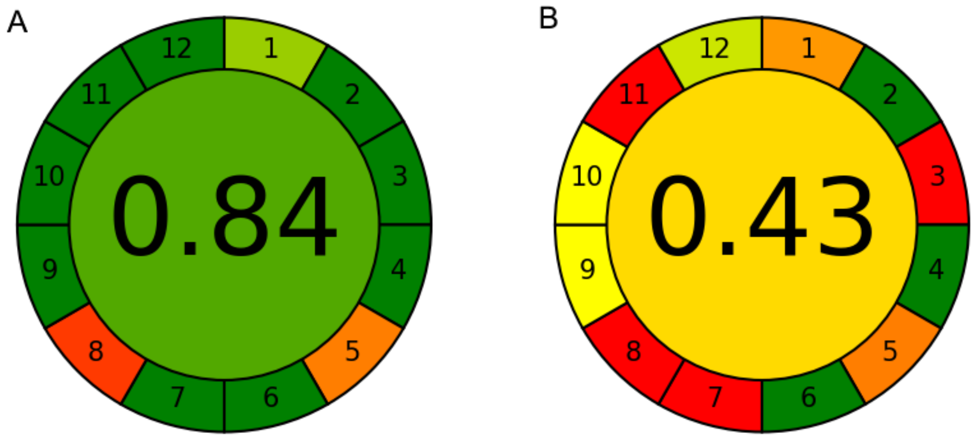

Figure S4. Results of evaluation according to the Analytical GREEnness metric approach for (A) in-line QCL-IR spectroscopy and (B) off-line HPLC analysis. The clock-like graphs comprise of 12 segments, representing the SIGNIFICANCE criteria. The performance of each principle is represented by a green-yellow-red color range, whereas the weight-importance is reflected by the width of each segment. The overall performance of the methods is expressed by the color and score (0=worst, 1=best) in the middle.

Table S1. Selected options for the Analytical GREEnness evaluation.

**Parameter**	**QCL-IR**		**HPLC**	
	**Selected option**	**Score**	**Selected option**	**Score**
1. Sampling procedure	On-line	0.7	Reduced number of steps	0.3
2. Amount of sample in g or mL	0.02	1.0	0.02	1.0
3. Position of analytical device	In-line	1.0	Off-line	0.0
4. Sample preparation steps	3 or fever	1.0	3 or fever	1.0
5. Automation, miniaturization	Semi-automatic, non- miniaturized	0.25	Semi-automatic, non-miniaturized	0.25
6. Derivatization	None	1.0	None	1.0
7. Amount of waste in g or mL	0	1.0	200	0.0
8. Number of analytes per run, sample throughput per hour	4, 0.5	0.12	4, 0.05	0.0
9. Most-energy intensive	FTIR	1.0	LC	0.5
10. Type of reagents	All are bio-based	1.0	Some are bio-based	0.5
11. Toxic reagents or solvents	0	1.0	60 mL	0.0
12. Threats	None	1.0	Toxic, flammable	0.6
